# Informal healthcare provision in Lebanon: an adaptive mechanism among displaced Syrian health professionals in a protracted crisis

**DOI:** 10.1186/s13031-019-0224-y

**Published:** 2019-08-28

**Authors:** Gladys Honein-AbouHaidar, Aya Noubani, Nour El Arnaout, Sharif Ismail, Hana Nimer, Marilyne Menassa, Adam P. Coutts, Diana Rayes, Lamis Jomaa, Shadi Saleh, Fouad M. Fouad

**Affiliations:** 1 Global Health Institute, American University of Beirut, Beirut, Lebanon; 2 Hariri School of Nursing, American University of Beirut, Beirut, Lebanon; 3 Department of Primary Care and Public Health, Imperial College London, London, UK; 4 Department of Sociology and Magdalene College, University of Cambridge, Cambridge, UK; 5 Syria Public Health Network, London, UK; 6 Department of Nutrition and Food Sciences, Faculty of Agricultural and Food Sciences, American University of Beirut, Beirut, Lebanon; 7 Department of Health Management and Policy, Faculty of Health Sciences, American University of Beirut, Beirut, Lebanon; 8 Department of Epidemiology & Population Health, Faculty of Health Sciences, American University of Beirut, P.O. Box 11-0236, Riad El Solh, Beirut, 1107 2020 Lebanon

**Keywords:** Refugees, Syria, Lebanon, Healthcare workers, Health system, Host communities, Informal provision

## Abstract

**Background:**

Syrian healthcare workers (HCWs) are among those who fled the Syrian conflict only to face further social and economic challenges in host countries. In Lebanon, this population group cannot formally practice, yet many are believed to be operating informally. These activities remain poorly documented and misunderstood by the academic, policy and humanitarian communities. This study aims to understand mechanisms of informal provision of services, the facilitators and barriers for such practices and to present policy recommendations for building on this adaptive mechanism.

**Method:**

A qualitative descriptive study based on an in-depth interview approach with a sample of Syrian informal healthcare workers (IHCWs) residing in Lebanon was adopted. Known sponsor networks followed by snowball sampling approaches were used to recruit participants. Data collection occurred between September and December 2017. All interviews were audio-recorded, transcribed and translated into English. An inductive thematic analysis was used.

**Results:**

Twenty-two participants were recruited. Motivational factors that led HCWs to practice informally were personal (e.g. source of income/livelihood), societal (cultural competency), and need to fulfill a gap in the formal health service sector. Being connected to a network of IHCWs facilitated initiation of the informal practice until eventually becoming part of a community of informal practice. The central challenge was the informal nature of their practice and its negative consequences. Most IHCWs were afraid of arrest by the government upon identification. Most interviewees indicated being discriminated against by host communities in the form of differential wages and tense interpersonal relationships. Almost all recommended a change in policy allowing them to practice formally under a temporary registration until their return to Syria.

**Conclusion:**

Our study confirmed the presence of IHCWs operating in Lebanon. Despite its informal nature, participants perceived that this practice was filling a gap in the formal health system and was helping to alleviate the burden of IHCWs and refugee health needs. In line with interviewees’ views, we recommend that policy decision makers within humanitarian agencies and the Government of Lebanon explore the possibilities for allowing temporary registration of displaced Syrian IHCW to benefit local host communities and refugee populations.

**Electronic supplementary material:**

The online version of this article (10.1186/s13031-019-0224-y) contains supplementary material, which is available to authorized users.

## Background

Formal employment in host community labour markets is a common challenge experienced by refugees across the globe, including skilled professionals such as healthcare workers (HCWs) [1–3]. Several underlying factors influence labour market integration for refugees. These include the perceived threat based on the absolute number of refugees compared to the host community [4]. The educational and skills level of the refugee population are seen as potential competitors, negatively impacting the structure and macroeconomic stability of the host community such as: increasing competition for jobs in an already fiscally constrained setting [5]. Fear of permanent integration, particularly in protracted crises, often lasts between 10 and 20 years. [4, 6]. Hence, the governments of most host communities restrict the formal participation of refugees in the local labour market leading many to work informally as an adaptive survival mechanism.

An informal sector, as defined by the International Labor Organization, occurs when employment is not covered or is insufficiently covered by formal arrangements [7]. Informality in health care is often defined in the literature as having no formal training, undocumented payment, and operates without formal regulatory functions [8]. In this study, IHCWs emerge when HCWs possess the core qualifications to provide healthcare but are prohibited by law and regulation from engaging in the formal sector [9]. Informal provision of public services such as health, education, food distribution is a prevailing phenomenon in refugee crises. According to Del Caprio and Wagner (2016), no matter what their qualifications, refugees tend to be employed in the informal sector [10].

Most of the evidence base focuses on the informal economies of low-skilled workers such as agriculture and construction [4, 10]. Recently, the focus on the high skilled workers such as HCWS in particular started to emerge [11]. The challenges of integrating HCWs into the workforce mirror those of other refugees in terms of being a threat to the local economy [12]. But more specifically, HCW face the challenge of lack of policies for assessing the equivalency of their professional credentials in crisis settings and the willingness to formulate such policies due to political reasons [13]. Thus leading many to practice informally but not without dire consequences, such as being arrested and deported [11]. Often, IHCW services are poorly documented and understood by the academic, policy and humanitarian communities and, little is known about the drivers for practicing informally despite the threat to their status as refugees or asylum-seekers in their host country.

The Syrian crisis is the largest refugee crisis since World War II. A key feature of the crisis has been the displacement of highly skilled workforce cadres – including HCWs – from a country that before the outbreak of conflict had a highly educated population in comparison to many regional neighbors [14–19]. Host countries, such as Lebanon, who received a large number of refugees [20] were poorly prepared on how to stipulate policies to manage this influx [21]. With limited fiscal understanding about refugee labour market integration, Lebanese policy makers restricted formal labour market opportunities to three domains: agriculture, construction and domestic cleaning services. Thus, limited policy consideration has been given to displaced highly skilled Syrians such as HCWs who attempt to practice in host communities [11, 17].

### Specificities of the context in Lebanon

The influx of Syrian refugees, coupled with coordinated efforts from a number of actors to mitigate financial access barrier to healthcare [22], led to an exponential increase in demand for healthcare services in Lebanon. To complicate the situation, the majority of refugees settled in mostly underserved areas already characterized by limited human resources and infrastructure [22–24]. Currently, the majority of health services provision is through primary healthcare centers (PHCs) and hospitals, both supported by the Lebanese Ministry of Public Health (MOPH), Non-Governmental Organizations (NGOs), and United Nation (UN) agencies for refugees registered with the United Nations High Commissioner for Refugee (UNHCR) [25]. They receive primary care consultations for a fee of 2–3 USD and pay 25% out-of-pocket of hospitalization fees [22, 26]. The exponential increase in demand without a corresponding increase in human resource capacity eventually led to inadequate access to health services for both Lebanese and refugees alike [15, 22, 27, 28]. It is estimated that 74% of Syrian refugees reported difficulty accessing formal healthcare, with some returning to Syria in order to receive services [22, 23, 29].

Previous research showed that IHCWs played a significant role in healthcare provision among poor and marginalized populations, including refugees, by filling the gap left by weak and overstretched formal institutions, such as the privatized hospitals and independent clinics [8, 30]. The main reported reasons for individuals seeking care from IHCWs were convenience, affordability, and social factors. The latter included being located in close proximity to refugee populations, thus reducing transportation costs for care seeking, generally having flexible working hours, accepting in-kind payment, and having an established reputation within the community, all of which strengthen their appeal to refugee communities [8].

Given the presence of HCWs among Syrian refugees and the inadequate access to the formal health sector, the emergence of an informal network of healthcare provision among refugees would seem a natural response mechanism. However, this adaptive mechanism of both refugees and HCWs is an under-explored area in humanitarian and protracted crises [23].

This exploratory study aimed to understand mechanisms of informal provision of services, the facilitators and barriers for such practices and to put forward policy recommendations for building on this adaptive mechanism based on the perspectives of Syrian HCWs providing care informally in Lebanon.

## Methods

### Study design

A qualitative descriptive study based on an in-depth interview approach with a sample of Syrian informal healthcare workers (IHCWs) residing in Lebanon was adopted.

### Ethics

Ethical approval was obtained from the Institutional Review Board at the American University of Beirut (AUB) before proceeding with the recruitment and data collection. Ethical approval was also sought and secured from the Centre for Business Research, University of Cambridge.

### Study participants

Male and female health workers of Syrian nationality, aged 18 years old above, and currently residing in Lebanon were included in this study. We used the maximum variation approach where we targeted HCWs from different specialties (such as medical doctors, nurses, physiotherapist and dentists), geographic variation and gender. Syrian health workers who had a dual citizenship and those who were practicing legally (if any) in Lebanon were excluded from the study.

### Sampling and recruitment

The legal and ethical considerations related to the topic of informal practice, in addition to the hard-to-reach nature of the target population in the absence of any official directory, necessitated the use of the “Known Sponsor” approach followed by the snowball sampling approach for recruitment [31, 32]. Under the “Known Sponsor” approach, members of the research team who knew an IHCW approached her/him inviting them to participate in the study. Those who consented participated in the first wave of interviews. Formal consent to participation was obtained orally at the beginning of each interview. Written consent was for the most part impossible to obtain from study participants – principally because of fears over personal status. At the end of each interview, the primary data sources (known sponsors) were asked to share in nominating another potential participant for the study.

### Data collection

A semi-structured approach for the interviews with predetermined, open-ended questions to guide the discussion was used for data collection (See Additional file 1). This approach was taken to ensure consistency between interviews and thus increase the reliability of findings [31]. However, the interviewer was prepared to depart from the planned itinerary if additional questions emerged throughout each interview [33] [34]. We translated the semi-structured interview guide (initially written in English) to Arabic, the native language of both the interviewee and interviewer, thus facilitating freedom in the expression of thoughts and avoiding misinterpretation due to language barriers [35]. Interviews were conducted in private spaces either at American University of Beirut or in health centers located in different regions across Lebanon, depending on the proximity of the location to the interviewer. All interviews were carried out in Arabic and audio-recorded upon consent of the participants, and each lasted on average 45 min. Saturation was reached upon completion of 23 interviews. However, one participant refused to be audio recorded and both the interview and the notes taken were not comprehensive enough; thus, 22 interviews were included in the final analysis. Participants’ confidentiality was observed by coding each audiotape and transcript.

### Data analysis

All semi-structured interview discussions were transcribed verbatim. An inductive thematic analytical approach was used to analyze the data [36]. Two researchers immersed themselves by reading the transcripts and formed an initial thematic framework for coding. Then, independently, they coded line-by-line the content of all transcripts. The codes were then compared and contrasted using the constant comparative approach and emerging themes were identified. Following several iterative approaches, the list of themes was subsequently refined and major final themes were agreed upon between the researchers. Findings were then reported in a thematic narrative approach, with examples of interviewees’ quotes included under the corresponding themes. The reporting of this study followed the consolidated Criteria for Reporting Qualitative research (COREQ) **(See** Additional file 2).

## Results

### Characteristics of the study population

22 in-depth interviews were conducted between September and December 2017. Characteristics of the participants are shown in Table 1. We included diverse health professionals, mainly males (*n* = 18) and majority from the Bekaa area (*n* = 14).

**Table 1:** Characteristics of the Study Population (*N* = 22)

Characteristics		N
Gender	Male	18
	Female	4
Practice location	Semi-urban	14
	Urban	8
Occupation	Medical Doctor	9
	Nurse	4
	Other healthcare professionals^*^	9
Years of professional experience in Syria	< 5	15
	5–10	4
	> 10	3
Years of professional experience in Lebanon	< 1	4
	1–2	6
	3–4	8
	> 5	4

Other = physiotherapist, pharmacist, dentist, psychologist, health administrators.

### Thematic findings

Five overarching themes emerged from the qualitative interviews including: 1) motivational ‘push’ factors to becoming IHCWs, 2) facilitators of building the IHCW community of practice, 3) challenges and implications of IHCW provision, 4) relationship with the host community’s healthcare system and funding mechanisms, and 5) recommendations suggested by the IHCW (Table 2).

**Table 2:** Describing the nature of IHCWs community of practice

Main Theme	Sub themes	Examples
Motivation	*Personal Factors*	➢ Altruism ➢ Source of livelihood ➢ Professional
	*Societal Factors*	➢ Gender congruency ➢ Cultural competency
	*Formal health services*	➢ Affordability ➢ Filling in a gap
Facilitators	*Networks enabling IHCWs to initiate work*	➢ Professional ➢ Family & friends
	*Building reputation among Syrian refugees*	
	*Establishing a community of practice*	➢ Social media ➢ Internal referrals among IHCWs
Challenges	*Personal Level*	➢ Constant threat ➢ Psychological wellbeing ➢ Mistrust ➢ Economic ➢ Continuous education
	*Societal Level*	➢ Resentment from the Lebanese community ➢ Competing with Lebanese providers
Implications	*Status in Lebanon*	➢ Detention/ deportation ➢ Losing residency status
	*Impact of care provided*	➢ Compromising patient services/closing centers
Relationship with the formal system and reimbursement mechanisms	*Government’s position*	➢ Legal threat, yet keeping a blind eye
	*Community-based NGO funded*	➢ Collegiate atmosphere ➢ Equity in wages ➢ Lebanese and Syrian patients
	*Hospital & pharmacies funded by Lebanese healthcare providers*	➢ Assistance to Lebanese HPs ➢ Less wages for Syrians ➢ Mainly Syrian patients
	*Linkage with formal practice*	➢ Referring patients to formal providers when necessary

### Motivational factors for informal practice

The study participants had to take considerable risks to become IHCWs in Lebanon, yet they opted to practice without registration and work permit for **personal and societal factors, and factors related to the formal health services.**

At the personal level, altruism, financial, and professional factors were highlighted. Interviewees stated that their main cue to practice was to alleviate the pain and suffering of their fellow refugees.

*“We are delivering a humanitarian service”*_*HP.2*_

Financial motivation was an equally important personal factor. With displacement comes unemployment and personal economic crisis. Hence, the ‘*Need for money*’ _(HP.1.O)_ motivated IHCWs to practice informally despite the legal challenges.

For some participants, preserving their professional skills was a major driving force to practice. IHCWs were resolute in continuing to practice despite the poor reimbursement and the legal challenges.

*“A doctor stays in his profession. We accept all the circumstances; being illegal, without permit in order not to leave the medical practice”*_*HP.2.B*_

At the societal level, gender congruence, i.e. females needing female ICHWs, was a factor leading one female health worker to practice informally.

*“I registered in the NGO for the purpose of receiving aid. They knew that I am an OB-GYN doctor and contacted me to work for the women's health center”*_HP.2.A_

IHCWs also perceive that they are valued because they are more culturally competent in working with refugees of Syrian origin*.* They indicated that IHCWs are linguistically competent, more empathetic, and more approachable than their Lebanese counterparts are.

*“Syrian patients feel more comfortable with us because we come from the same culture”*_*HP.1.A*_

Factors related to pursuing ***formal health services*** focused on affordability of IHCW services, where local patients compared the IHCW’s (often-gratuitous) consultation fee to that of the more expensive Lebanese healthcare providers. Hence, the IHCW was more in demand.

*“The patient, whether Syrian or Lebanese, only cares for the offered health provision. This service is offered for symbolic fees”*_*HP.1.B*_

Some pointed out that the formal healthcare system was burdened by an excessive demand for health services, and that IHCWs were therefore filling in a gap in existing service provision. They perceived themselves as the right people, in the right time and place to provide health services in rural parts of Lebanon that lack access to health professionals and specialized health services.

*“Syrian doctors are trying to provide services in areas that lack physicians and appropriate medical services” HP.2*

*(See* Additional file 3*)*

### Facilitators for becoming IHCWs

Participants described the process that facilitated becoming an IHP. First, they were connected to a network of IHCWs in Lebanon. Then they gradually started to build a reputation among communities of Syrian refugees until they eventually gained traction and became part of an informal community of practice where IHCWs got connected and formed a network of practice.

Concerning the initiation of this informal work, some participants indicated that they started contemplating the idea of becoming an IHCW before they were displaced to Lebanon having exploring their opportunities with fellow Syrian healthcare professionals who were already displaced and already practicing informally in Lebanon.

*“Dr. [..] Once came to Syria. I knew him and he knew me. Once I came, he immediately contacted me, and started working with them”*_*H.P.1.D*_

Other participants indicated that as soon as they were displaced, they were connected through friends, family, or colleagues, as well as international and local NGOs who helped them start. A few started working *“as soon as [they] arrived in Lebanon”*_HP.1.A_, while others had up to *“4 months”*_*H.P.1.B*_ before they started working.

As soon as they started working, they built their professional reputation among Syrian refugees through word-of-mouth. That is, once IHCWs started working, they become better known within the Syrian community regarding their skills and services, and subsequently became known in their respective geographic areas:

*“So they [the patients] tell me: people gave me your contact number to reach me”*_*HP.1.O*_

For some, gaining traction to become part of IHCW community of practice occurred organically when they were introduced to other IHCWs within their workplace. The IHCWs from different professions and specialties clarified that they built a network of communication through social media, such as Facebook and WhatsApp, to facilitate the referral process of the Syrian patients to the proximate and suitable health facilities:

*“We (The Syrian doctors in Lebanon) built networks of communication. The main aim of this network is to refer patients to the appropriate health services”*_*HP.2*_

*“The connections are being [made] through social media (Facebook)”*_*HP.1.L*_

*(See* Additional file 3*)*

### Challenges and implications

#### Challenges

Practicing informally imposed several challenges to the personal and social aspects of IHCWs’ livelihoods. At the personal level, IHCWs who participated in this study indicated that their biggest challenge was the ‘informal’ practice itself, which resulted in a stigma associated with working illegally as one participant indicated:

*"You need to consider that our work is illegal, hence you [as a provider] would directly feel uncomfortable and stressed out."*_*HP.1.L*_

Also, participants stated that they were under *“continuous feeling of threat”*_HP.1.K_ for being reported and prosecuted, which affected their daily lives and psychological well-being. Some even suspected that their patients may be reporting them, so they could not *“trust all [their] patients”*_*HP.1.L*_*.*

Financial inequality and discrimination based on nationality where the “*salary is less than half the salary of a Lebanese worker”*_*HP.2.A*_ was also reported as a challenge with tangible, negative repercussions on the IHCWs well-being.

These economic challenges also prevented some IHCWs from participating in continuing their professional education:

*“I am thinking of applying to do a master’s degree, but because of my relocation and moving to Lebanon, I couldn’t continue my education”*_*HP.1.K*_

At the societal level, IHCWs sense that the host community typically resents the presence of Syrian refugees:

*“The Lebanese citizen would ask me: ”you are Syrian, what are you doing in our country?*_*HP.2.C*_

In addition, IHCWs believe that the Lebanese healthcare professionals perceive that Syrian health workers are competing for their clientele:

*“Lebanese doctors consider that we took their patients away and we are competing with them”*_*HP.1.K*_ *.*

#### Implications

The participants reported several implications for their work as IHCWs. Some revealed being reported to the government and detained, but eventually released:

*“Most of those I know faced legal issues. Some of the doctors that were known to be working within their specialty were deported from the country within a week”*_*HP.1.G*_

Many participants reported their inability to renew their residency permits because of their informal and unregistered practice; rendering them illegal residents in Lebanon, living in continuous fear of being detained or deported.

*“There were major challenges in the renewal of residence and especially that we don’t have any work permit. The obstacles at work were mainly the legal issues with the work permit. There is a continuous feeling of threat”*_*HP.1.K*_

*“We went to someone as a facilitator to work on my residency in Lebanon. He took 600 USD and after 6 months he could not do anything and I didn’t get my money back. I don’t want to put myself in a situation where I might be stopped by the police men and undergo investigations for not being legal.”*

HP.2.C.

Others stated the challenges of maintaining a proper work permit in Lebanon, which may involve exiting the country often in order to remain legal:

*“I was deported from the country. I lost my work permit in Lebanon and that’s why I stay in the country for a month and I should leave for a month”*_*HP.1*_

Others reflected on the threat of closing the centers where they practice, stressing that not only will IHCWs lose their jobs, but patients’ needs will no longer be met.

*“Our fear at the center is to have it closed for any reason: legal, medical…etc. we are afraid because it is a huge loss for the patients who are receiving their treatments. There is almost 200 operations per month and around 3000 during 2017 and more than 1800 checkups”*_*HP.2.C*_

*“After one and a half day, the army attacked the center and major problems happened.”*_*HP.1*_

*(See* Additional file 3*)*

#### Relationship of IHCWs with the host Community’s healthcare system and funding mechanisms

A few IHCWs indicated that the Lebanese government is aware of their informal services but is *“keeping a blind eye”*_HP.1.D_ and attributed this to the dire need for services in areas where most Syrian refugees are situated; areas that are already under-resourced. However, most participants were concerned that *“once the government points to someone who is practicing illegally, the situation will be very bad”*_(HP.3.A)_.

IHCWs practice in several locations, including community-based centers, hospitals, and clinics. IHCWs working in community-based centers such as primary healthcare centers (funded or run by NGOs), work under the cover of the corresponding governing NGO. Those IHCWs work alongside Lebanese health workers offering similar services to the same population of patients. IHCWs working in such settings described the environment as collegial with equal monetary reimbursement among Syrian and Lebanese health workers.

*“I don't work alone; there is a Lebanese [specialist] who is present, because the center is Lebanese. I only communicate with a Lebanese [specialist]: I transfer some cases to her and she does the same with some cases to do it with the UN. She usually sees them, the Syrian patients. She transfers them to me and in return, sometimes I have patients who are not covered by UN, she charges less fees to help them as well.”*_*HP.1.O*_

Other IHCWs work in hospitals funded by Lebanese health workers. Although they work side by side, IHCWs are often portrayed as assisting their Lebanese counterparts, thus are less remunerated. In such settings, patients admitted to the hospitals are assigned to Lebanese health workers on paper, while in reality, the service is often provided by the IHCWs.

*“As an ER doctor, I check up on the patients and refer them to the appropriate doctors. I work under the coverage of a Lebanese doctor and I am always in contact with him. He covers me, I write my prescriptions under his name. I follow up with him and inform him with every step.”*_*HP.2.C*_

*“The center is controlled and covered by Lebanese doctors and we act as their assistants”*_*HP.1.K*_

Often, Syrian patients are cared for by IHCWs, as their consultation fees are less costly. However, if the service cannot be provided by an IHP, then they are referred to a Lebanese health worker and they negotiate cheaper fees, advocating for refugee patients.

*“We refer patients to Lebanese doctors, but mainly we inform the doctor that the Syrian patient won’t be able to afford paying as the Lebanese patient”*_HP.1.J_.

Syrian IHCW pharmacists and physical therapists also reported working undercover in private offices of Lebanese health workers, for a minimal salary.

*“[I] started in a [Lebanese] pharmacy with a very low salary”*_*HP.1.C*_ *.*

Finally, IHCWs prefer to refer patients within their own community of practice due to lower financial costs. However, if the service is not available within the informal community, then they refer them to formal Lebanese doctors.

*“The critical cases that we are not able to deal with are always referred to the Lebanese doctors”*_*HP.1.K*_ *.*

*(See* Additional file 3*)*

#### Recommendations as suggested by IHCWs

To improve their status, most participants suggested that the Lebanese government should acknowledge the contribution of the IHCWs to the Lebanese healthcare system through responding to the health needs of the Syrian refugees residing in the country, consequently alleviating the burden faced by the system by filling existing gaps in resources. The majority of participants requested that the government registers IHCWs, even if temporarily, in order for them to practice legally until they eventually return to Syria. They also asked for opportunities for continuous education.

*“If we can get a work permit such that the Syrian doctors can work with Syrian refugees only”*_*HP.2.A*_

*“We can help by reducing the burden on the Lebanese health system”*_*HP.2.A*_

## Discussion

Our results confirm the presence of an informal network of healthcare provision among Syrian refugees in Lebanon. To our knowledge, this study is one of the first studies to document informal health service provision of this kind for refugee populations in a low- and middle-income setting in the context of protracted crisis. The majority of interviewees undertook their practice in areas where the highest number of refugees is concentrated, in impoverished areas and where health services are most needed [22–24]. IHCWs were engaged in informal practice for personal, professional, financial, social and altruistic reasons – including helping to fill a gap that they recognized in service provision through the formal system. They faced various challenges but the greatest risks highlighted by participants stemmed directly from their status as practitioners operating without local registration and accreditation in Lebanon. To improve their working conditions, IHCWs recommended formalizing their practice by allowing temporary registration until their return to Syria.

Our findings regarding challenges accessing the formal Lebanese healthcare system are supported by those in Parkinson and Behrouzan (2015) who found that between 55% and 74% of Syrian refugees experience difficulty accessing healthcare across Lebanon [23]. Furthermore, the fact that affordability and cultural competency, along with convenience, were important factors leading Syrian refugees to seek IHCW care echo those reported in a systematic review examining the various reasons individuals seek informal healthcare [8].

The definition of informal healthcare provision revealed by participants in this study has similarities with is described elsewhere in the literature on this topic, but also important points of difference. Much of the existing literature on informality in healthcare provision originates from low or lower-middle income country contexts, where “informal” health workers are understood as having received training outside a formal institutional setting or curriculum, receive undocumented payment, operate without oversight and may be members of professional bodies that do not have formal regulatory functions [8, 37, 38]. The participants in this study, however, do not meet the first criterion in this definition: they possess formal qualifications from Syria, and their informal status derives instead from legal, regulatory, administrative or other barriers to registration in Lebanon.

The fact that IHCWs are part of a larger community of informal workers, who promote each other’s services through referrals and the use of social media to connect is also in line with the broader literature on informality in healthcare, which indicates that the work of informal healthcare workers is always tied to the community and is dependent on their trust [8]. We found that IHCWs deliver services to Syrian refugees throughout all health sectors, including public, private, and humanitarian [22]. However, work conditions are diverse, with NGOs providing equal pay while the Lebanese-run private hospitals and pharmacies provided differential wages between IHCWs and local Lebanese health care workers. This inequity is widely present across the labor market, and is not only restricted to the health sector, where Syrian refugees are being paid lower wages than their Lebanese counterparts, despite longer working hours and the persistent lack of social benefits [24].

Informal healthcare systems emerge when and where formal institutions are unable to cope, often within marginalized communities [8, 30]. Therefore, informal mechanisms are not inherently at odds with formal institutions, but instead try to fill a gap. In the case of Syrian refugees there is a massive deficiency in access to healthcare services [22, 23]. Moreover, even when services are accessible, they are often too costly for refugees, another issue that is currently being addressed by the informal workforce as evidenced by our results [22, 24, 29]. Moreover, a high prevalence of NCDs among the refugee population requires continuity of care and resourcing that the mainstream Lebanese healthcare system cannot provide [27]. The IHCWs have shared that they were instrumental in filling gaps in the formal system and addressing the health needs of Syrian refugees.

Despite the benefits from their services, the informal nature of their work was perceived by IHCWs as the greatest challenge they face. IHCWs work in stressful and dangerous situations. The majority of the study participants reported concern that the Lebanese government will crack down on their practice, which may lead to severe consequences. Some of the interviewed individuals reported being personally reported, detained, or witnessed colleagues suffering these consequences. Syrian refugees in similar situations opted to leave Lebanon, thus creating a vacuum in services currently needed in-country [39]. And the fear is that in the long run, a large refugee population will be left with limited access to healthcare [39].

The laws in Lebanon prevent non-Lebanese physicians from practicing medicine, and such labor restrictions are also present in other host countries [11, 12] Worldwide, the urgency of the refugee crisis prompts host countries to consider first providing access to basic needs, including health. In a protracted situation, the focus is often adapting mechanisms such as language literacy and other social services. Ultimately, employment and integration policies are drafted but the spectrum of integrating refugees in the workforces varies widely between countries and they range between two extremes and everything in between. For example, Sweden is generous with social policies but very restrictive in work integration while the US is restrictive in social policies and encourages rapid integration [13]. In the case of the Syrian crisis, Lebanon, like Sweden, was very restrictive due to the limited fiscal understanding of refugee labour market integration on one hand [10, 16] as well as the the fear of permanent integration of refugees, especially in a protracted crisis situation that can often last between 10 and 20 years [4, 6]. Although the Syrian crisis has had a net negative impact on the economy, the Lebanese government can ease this impact by increasing flexibility in labour market regulations.

The United Nations Development Program (UNDP) issued a report, Jobs Make the Difference [39] where the two main requirements for boosting economy with co-benefit for both refugees and host communities are: 1- strong political will to pass reforms such as increasing flexibility in labour market regulations; 2- paired with increasing multiyear funding from developed countries where the funds are blended between humanitarian and developmental resources Lebanon can also learn from other host communities such as Turkey, Egypt and Germany. The Turkish government, faced with a dire need for health workers, also within a domestic climate in which political commitment to the goal of universal health coverage is apparently strong, recently provided Syrian physicians and other health workers with temporary registration allowing them to practice among their communities [13, 17, 39–43]. Egypt has also removed some restrictions, allowing Syrian physicians, nurses, and midwives to provide healthcare to other Syrians [39]. These initiatives not only provided healthcare workers with opportunities for employment, but also created a much-needed healthcare workforce. Germany took a different approach by providing Syrian doctors short-term visas, bypassing the long asylum application process. The healthcare gap in Germany is of a different kind, due to a general shortage of physicians and a rapidly aging population. [44] As a result, there are nearly 1500 Syrian physicians now working in Germany to fill this gap [44].

The participants in this study mentioned that they sense the tension created by their practice, perceiving that Lebanese physicians feel that they are taking over their livelihood. Lebanese doctors have complained to the syndicate of doctors about the illegal practice of some IHCWs, leading to their detention and termination of practice [11, 13]. While restricting the work of Syrian doctors to serve their community may avoid competition with Lebanese healthcare workers, they can also serve as a means to decrease the load on overworked Lebanese healthcare workers and a strained healthcare system.

### Policy recommendations

Despite being a qualitative study capturing the perspective of a small sample of informal provider of services, we believe that the importance of the findings of this study still reflect the current context of informal provision of services and its various challenges. Hence, we second the opinion of our participants and recommend that the Lebanese government consider issuing short-term licenses for Syrian health workers, which would allow them to provide healthcare to their compatriots [26, 45]. It will serve the health workers on a personal level by providing them with formal employment and a legal source of income [39, 45]. The main argument for adopting such as policy would be to complement the formal health care system rather than substituting or competing with it [30]. This policy will also integrate the advantages of informal healthcare provision, mainly affordability and cultural competence, into a formal regulated system. Moreover, it will decrease the patient load on the primary healthcare centers and other healthcare workers in the most overcrowded and understaffed areas [45]. It will also be imperative to address the dominant fear of permanent integration of refugees as a barrier to integrating Syrian healthcare workers in Lebanon. Therefore, it is recommended to clarify that such a policy does not desire to provide permanent integration of IHCWs into the Lebanese health care workforce; rather, it is a mean of improving their current living conditions [30] and allowing them to support and contribute to the currently overburdened national healthcare system. Finally, it will allow healthcare workers to retain their professional skills in order to help re-build the Syrian healthcare workforce [13, 15, 16, 39].

For a limited registration model to operate effectively, medical knowledge and skills need to be assessed and equated with the host community standards. However, it should be noted that many essential documents required for equivalency may be unavailable to the health workers, due to the difficulty of obtaining them from the Syrian government and Syrian medical institutions [46]. The Lebanese authorities could consider adopting the strategy followed by Turkey where a short training course in universities will issue a certificate that will act as an assessment method for obtaining a license [13]. Special consideration may be needed for senior medical students and junior doctors who were also affected by displacement, and were therefore unable to complete their training [46]. It is also essential to support the issues of this population and aid them in proceeding in their education in order to receive a qualifying license.

### Limitations & strengths

This study tackled informal work, and as such participants might have been uncomfortable disclosing information that was too personal or that could jeopardize their informal practice. Further, the informal nature of work acted as a barrier to recruitment, with no official registries or institutions to access information from. In particular, the snowball-based approach to recruitment inevitably raises the risk of selection bias – although we considered it the most pragmatic means of accessing this particularly hard-to-reach population. The participant sample was also predominantly male, suggesting a further source of selection bias. Despite these limitations, we were able to recruit key individuals with rich experience in IHCWs that formed the basis of this first study of its kind addressing the informal healthcare workforce in Lebanon. Another limitation of this study is that it captured the perspective of IHCWs only. The perspectives of SRs on one hand and those of the Lebanese health care professionals on the other hand are needed in order to triangulate the findings and get a comprehensive perspective on this topic.

## Conclusion

This study confirmed the presence of a significant informal healthcare workforce operating among Syrian refugees. These individuals are distinguished from conventional definitions of health workforce informality by the fact that they are all qualified practitioners – but lack the local registration and accreditation in Lebanon required to operate legally. This workforce fills the gap created by formal institutions and is essential for the provision of healthcare services to Syrian refugees. There is a pressing need for policy measures to integrate this workforce through official channels by providing them with temporary licensing that will allow them to provide healthcare services to other Syrian refugees until their return to Syria. This will, in turn, alleviate some of the burden on the Lebanese healthcare system, while avoiding competition with the host community.

## Additional files

Additional file 1: Topic Guide for the Qualitative Interview: ‘Informal’ Provision of Health Services for Syrian Refugees in Lebanon. (DOCX 20 kb)

Additional file 2: Consolidated criteria for reporting qualitative studies (COREQ): 32-item checklist. (DOCX 15 kb)

Additional file 3: Additional Quotes. (DOCX 24 kb)

## Data Availability

The data generated and analyzed during the current study are not publicly available due to confidentiality concerns but are available from the corresponding author on reasonable request.

## Abbreviations

AUB: American University of Beirut
HCWs: Healthcare workers
IHCWs: Informal healthcare workers
MOPH: Ministry of Public Health
NGOs: Non-Governmental Organizations (NGOs)
PHCs: Primary Healthcare Centres
UN: United Nations
UNHCR: United Nations High Commissioner for Refugee
