# Correction to: Informal healthcare provision in Lebanon: an adaptive mechanism among displaced Syrian health professionals in a protracted crisis

**DOI:** 10.1186/s13031-019-0229-6

**Published:** 2019-10-08

**Authors:** Gladys Honein-AbouHaidar, Aya Noubani, Nour El Arnaout, Sharif Ismail, Hana Nimer, Marilyne Menassa, Adam P. Coutts, Diana Rayes, Lamis Jomaa, Shadi Saleh, Fouad M. Fouad

**Affiliations:** 1 Global Health Institute, American University of Beirut, Beirut, Lebanon; 2 Hariri School of Nursing, American University of Beirut, Beirut, Lebanon; 3 Department of Primary Care and Public Health, Imperial College London, London, UK; 4 Department of Sociology and Magdalene College, University of Cambridge, Cambridge, UK; 5 Syria Public Health Network, London, UK; 6 Department of Nutrition and Food Sciences, Faculty of Agricultural and Food Sciences, American University of Beirut, Beirut, Lebanon; 7 Department of Health Management and Policy, Faculty of Health Sciences, American University of Beirut, Beirut, Lebanon; 8 Department of Epidemiology & Population Health, Faculty of Health Sciences, American University of Beirut, P.O. Box 11-0236, Riad El Solh, Beirut, 1107 2020 Lebanon

**Correction to: Confl Health**

**https://doi.org/10.1186/s13031-019-0224-y**

The original publication of this article [1] contained an incomplete funding section. The full funding section is listed in this correction article. The missing information is indicated in bold.

**Funding**

This study was funded by the Collaborative Research Stimulus at the American University of Beirut, Lebanon, and by an accountable grant from the International Institute for Environment and Development (IIED) in the UK [grant no. 719.10/42, and **through the UK Research and Innovation GCRF RESEARCH FOR HEALTH IN CONFLICT (R4HC-MENA); developing capability, partnerships and research in the Middle and Near East (MENA) ES/P010962/1.**
