# Supplementary material for: Informal healthcare provision in Lebanon: an adaptive mechanism among displaced Syrian health professionals in a protracted crisis
Source: Confl Health. 2019 Aug 28;13:40. doi: 10.1186/s13031-019-0224-y (PMC6714409; doi:10.1186/s13031-019-0224-y)
Supplement: Supplementary file 1 — Topic Guide for the Qualitative Interview: ‘Informal’ Provision of Health Services for Syrian Refugees in Lebanon. (DOCX 20 kb) [file 13031_2019_224_MOESM1_ESM.docx]

**My name is .. . I am interested in learning more about the impact of displacement on Syrian health professionals and their role within health provision in Lebanon. Please note that all your answers will be treated with confidentiality and anonymity and will not be shared.**

**Introduction**

You recently indicated during an interview with one of our team that you are a health care professional trained in Syria and currently residing in Lebanon.

***Opening question***

Can you please tell me what your healthcare profession is (for example doctor, nurse, midwife, pharmacist etc.)?

**Further questions**

For how long did you practice in Syria?

Would you please briefly share some background about your professional experience? What is your main area of expertise? Who was your patient population in Syria?

When did you arrive in Lebanon and for how long have you been working in Lebanon? Describe to me your professional experience after being displaced to Lebanon?

If you have stopped working in healthcare, what have you been doing? How are you keeping up with your professional knowledge? Are you still in contact with some of your patients?

***Questions related to the types of health services currently being involved in***

***( The participant should not identify by name the people or organizations that assist them)***

Refugees living in Lebanon may face challenges accessing health care services. Can you share the type of health services you are currently providing to your local community? What challenges have you faced doing this?

**Further questions**

Are some of your previous patients from Syria residing in your local community? If yes, do they come to you seeking health care informally? What type of health services do you provide to them?

Do other individuals living in your current local community know you are a trained healthcare professional? If yes, how do they know? Do they ask for your service? What type of health services do you provide them with?

Do you see them in your own residence? Do you carry out home visits? Do you have access to medical equipment to assist in the provision of care? Do you have someone who assists you?

***Questions related to informal provision of health services***

Are you concerned about the legality of practicing in a country where you are not formally licensed to practice? Can you describe your concerns in more details please?

**Further questions**

Have you encountered any problems so far? If yes, what was the outcome?

***Questions related to the functionality of these health services***

Are the local community appreciative of your services?

**Further questions**

Did they tell you their views?

Did you note a difference in their health situation after your provision of health service?

How about your peer professionals, do you get the chance to interact with them? Do you share your concerns and views about current interventions? Describe the **kind of interaction** you have? Do you get the sense that they support you? Deter you?

***Questions related to connections to the formal health system***

Do you interact with your peers practicing formally in Lebanon? What was the nature of these interactions? Were there any particularly challenges?

**Further questions**

Are you in contact with those residing in your local community? Or outside your local community?

Do they know that you are informally providing health services to your fellow Syrians?

What is their attitude towards this informal provision?

If they have a Syrian refugee who had an access barrier to their services, do they refer them to you for care? Describe to me one situation where you provided a service from someone referred to you from the mainstream professional.

If you have a Syrian refugee who needs a service that you cannot provide, how do you refer them to a registered health professional to receive appropriate care? Do you encounter any barriers during the referral process? Tell me more about this experience?

***Recommendations***

Do you have **any suggestions for improving the potential role informal health care providers can make to alleviate the access burden to services among Syrian refugees? What about the role in reducing the burden on the host community health care system?**

Is there **anything you would like to add**?
