# Supplementary material for: Informal healthcare provision in Lebanon: an adaptive mechanism among displaced Syrian health professionals in a protracted crisis
Source: Confl Health. 2019 Aug 28;13:40. doi: 10.1186/s13031-019-0224-y (PMC6714409; doi:10.1186/s13031-019-0224-y)
Supplement: Supplementary file 3 — Additional Quotes. (DOCX 24 kb) [file 13031_2019_224_MOESM3_ESM.docx]

| **Themes** | **Sub-themes** | ***Exemplary quotes*** |
| --- | --- | --- |
| Motivation | **Personal**  Altruism  Financial  Professional | *“It is a humanitarian thing. Is it possible to see someone in an accident and not offer help? “_(_*_HP.1.A.)_.  *Need for money*’ _(HP.1.O)_  “*I insisted on staying in this profession although I had some opportunities to work as an accountant, which I did not take and started in the pharmacy with a very low salary”_HP.1.C._*. |
|  | **Societal**  Gender congruence  Cultural competency | *“I registered in an NGO for the purpose of receiving aid. They knew that I am an OBGYN doctor and contacted me to work for the women's health center”* _(HP.2.A)_.  *“We have the same culture and traditions. This makes him [the patient] feel comfortable”* _(HP.2)._ |
|  | **Formal health services**  Affordability  Filling in a gap | *“Here, the biggest surgery costs $1,000 so maybe because of that since it is much cheaper than other hospitals”* _(HP.1.O)._  *“Syrian doctors are trying to provide services in areas that lack physicians and appropriate medical services”* _(HP.2.)._ |
| **Facilitators** | **Networks**  Professionals  Family and friends | *My friend introduced me to IHP when they were visiting [city in Syrian]. When they knew that I was around, they welcomed me to their team* _(HP.1.N)_  *“Via people who come here; Anyone who comes here, we ask him to tell his family and Syrian friends. Me as a volunteer, I took advantage of this opportunity”_(HP.1.M)_* |
|  | **Building reputation among SRs** | *“The people knew about a [specialty], who came to the Bekaa area, I started receiving phone calls from patients and went to their homes to treat them.” _(HP.1)_* |
|  | **Establishing a community of practice**  Social media  Internal referrals among IHPs | *“There is a Facebook group and a WhatsApp group. We always send closed messages.” _(HP.1.K)_*  *“We built networks of communication, through Facebook group. The main aim from this group is to refer patients to the appropriate health services based on geographical locations and specialties. We also have a WhatsApp group for Syrian doctors.” _(HP.2)_* |
| **Challenges and implications** | **Challenges**  Personal  Constant threat  Psychological well-being  Mistrust  Economic  Continuous education  Societal  Resentment from the Lebanese community  Competing with Lebanese providers | *“You are always threatened of losing our source of living.” _(HP.1.G)_*  *“There is always stress. The ministry of health can come and inspect the center any time.” _(HP.1.J)_*  *“I am afraid. I no longer talk over the phone and I no longer have internet connection. I make sure who is the person I am talking to because I was previously exposed to the detention and investigation.” _(HP.3.A1)_*  *"We the Syrians are not being treated as the Lebanese doctors, because we are not allowed to work. We work in the afternoon like volunteers. We have minimal wages and that’s why we work in several centers.” _(HP.2.A)_*  *“Honestly, I used to love my job. There was a workshop at AUB on conflict medicine. It was very expensive, around 1500 USD. I couldn’t register because the fees were expensive _(HP.2.)_*  *“That patient that I was treating harmed my car's tiles. When people know that you are a Syrian refugee, many problems start to happen with you.” _(HP.1.I)_*  *“The Lebanese doctors consider that we took their patients away and we are competing with them” _(HP.1.K)_* |
|  | **Implications**  **Status in Lebanon**  Detention/deportation  Loosing residency status  **Impact of care provided** | *“The story started when the surrounded hospitals discovered that there was a Syrian hospital that is delivering services. The Lebanese government closed this hospital and arrested all the Syrian health workers because of the illegal practice. The whole Syrian health care workers were arrested….The staff were arrested for two days and then released after signing a pledge to never work again in the health field in Lebanon” _(HP.1.L)_*  *“Legally, you are only allowed to work within three categories: farmer, concierge, and construction builder….On my passport, my profession is written as doctor so it was a negative thing for my residency in Lebanon” _(HP.1)_*  *“After one and a half day, the army attacked the center and major problems happened.” _HP.1_* |
| **Relationships with the formal system and reimbursement mechanisms** | Government’s position  **Community-based NGO funded**  **Hospital & pharmacies funded by Lebanese HPs**  Assistance to Lebanese HPs  Less wages for IHPs  Mainly Syrian patients  Linkage with the formal practice | *“The Lebanese government gives a blind eye about the Syrian IHPs]” _(HP.1.D.)_*  *“There are Lebanese doctors here in the center. The center is not 100% Syrian. The manager is Lebanese, and there are 4 Lebanese doctors. They get Lebanese patients, and treat Syrian patients. There is a collaboration of efforts, resources, and expertise. Something very organized” _(HP.1.H)_*  *“He [Lebanese physician] covers me, I write my prescriptions under his name. I follow up with him and inform him of every step” _(HP. 2.C)_*  *“I had to apply to this pharmacy and the salary was very low but at least it is something”* _(HP.1.C)_  *“ I only communicate with a Lebanese [specialty]: I transfer some cases to her and she does the same with some cases to do it with the UN. She usually sees them, the Syrian patients. She transfers them to me and in return, sometimes I have patients who are not covered by UN, she charges less fees to help them as well.”* *_(HP.1.O)_*  *“So the big surgeries, we cannot cover here, we don't have an operating room. We transfer to other hospitals with a reduced price. We negotiate with the hospital to give them a reduced price but we cannot pay for them” _(HP.1.D)_* |
| **Recommendations** | Policy allowing them to practice formally under a temporary registration until their return to Syria  Opportunities for continuous education | *“The Syrian physician had studied long years and he came to Lebanon as someone who has worked hard on himself. The Lebanese government can make use of these human resources and to allow the Syrian providers to deliver services to the refugees who are in need. The benefits will be mutual.”_(HP.2)_*  *“I wish my situation gets legal and formal. I hate being an illegal person” _(HP.2.C)_*  *“If we want to develop our skills and capacity at the center, training sessions and workshops every once in a while (5-6 months) in the field of physiotherapy should be organized. This would develop our skills and knowledge for better service deliver.”_(HP.1.)_* |
